# What are the benefits and harms of risk stratified screening as part of the NHS breast screening Programme? Study protocol for a multi-site non-randomised comparison of BC-predict versus usual screening (NCT04359420)

**DOI:** 10.1186/s12885-020-07054-2

**Published:** 2020-06-18

**Authors:** David P. French, Susan Astley, Adam R. Brentnall, Jack Cuzick, Richard Dobrashian, Stephen W. Duffy, Louise S. Gorman, Elaine F. Harkness, Fiona Harrison, Michelle Harvie, Anthony Howell, Andrew Jerrison, Matthew Machin, Anthony J. Maxwell, Lorna McWilliams, Katherine Payne, Nadeem Qureshi, Helen Ruane, Sarah Sampson, Paula Stavrinos, Emma Thorpe, Fiona Ulph, Tjeerd van Staa, Victoria Woof, D. Gareth Evans

**Affiliations:** 1 Manchester Centre of Health Psychology, Division of Psychology and Mental Health, School of Health Sciences, University of Manchester, Coupland Street, Manchester, M13 9PL England; 2 NIHR Manchester Biomedical Research Centre, Manchester Academic Health Science Centre, Central Manchester University Hospitals NHS Foundation Trust, Manchester, England; 3 Division of Informatics, Imaging and Data Sciences, School of Health Sciences, University of Manchester, Manchester, England; 4 Centre for Cancer Prevention, Wolfson Institute of Preventive Medicine, Queen Mary University of London, London, England; 5 East Lancashire Hospitals NHS Trust, Royal Blackburn Hospital, Haslingden Road, Lancashire, BB2 3HH England; 6 The Nightingale and Prevent Breast Cancer Centre, Manchester University NHS Foundation Trust, Manchester, M23 9LT England; 7 NIHR Greater Manchester Patient Safety Translational Research Centre, University of Manchester, Manchester, M13 9PL England; 8 Patient representative, Manchester, England; 9 Manchester Breast Centre, Manchester Cancer Research Centre, University of Manchester, 555 Wilmslow Rd, Manchester, M20 4GJ England; 10 Department of Medical Oncology, The Christie NHS Foundation Trust, Wilmslow Rd, Manchester, M20 4BX England; 11 Research IT, IT Services, University of Manchester, Manchester, M13 9PL England; 12 Division of Population Health, Health Services Research & Primary Care, School of Health Sciences, University of Manchester, Manchester, M13 9PL England; 13 School of Medicine, University of Nottingham, University Park, Nottingham, NG7 2RD England; 14 Genomic Medicine, Division of Evolution and Genomic Sciences, The University of Manchester, St Mary’s Hospital, Manchester University NHS Foundation Trust, Oxford Road, Manchester, M13 9WL England

**Keywords:** Screening, Breast cancer, Risk stratification, High risk, Psychological impact, Early detection, Mammographic density, Chemoprevention, Tyrer-Cuzick, Anxiety

## Abstract

**Background:**

In principle, risk-stratification as a routine part of the NHS Breast Screening Programme (NHSBSP) should produce a better balance of benefits and harms. The main benefit is the offer of NICE-approved more frequent screening and/ or chemoprevention for women who are at increased risk, but are unaware of this. We have developed BC-Predict, to be offered to women when invited to NHSBSP which collects information on risk factors (self-reported information on family history and hormone-related factors via questionnaire; mammographic density; and in a sub-sample, Single Nucleotide Polymorphisms). BC-Predict produces risk feedback letters, inviting women at high risk (≥8% 10-year) or moderate risk (≥5 to < 8% 10-year) to have discussion of prevention and early detection options at Family History, Risk and Prevention Clinics. Despite the promise of systems such as BC-Predict, there are still too many uncertainties for a fully-powered definitive trial to be appropriate or ethical. The present research aims to identify these key uncertainties regarding the feasibility of integrating BC-Predict into the NHSBSP. Key objectives of the present research are to quantify important potential benefits and harms, and identify key drivers of the relative cost-effectiveness of embedding BC-Predict into NHSBSP.

**Methods:**

A non-randomised fully counterbalanced study design will be used, to include approximately equal numbers of women offered NHSBSP (*n* = 18,700) and BC-Predict (*n* = 18,700) from selected screening sites (*n* = 7). In the initial 8-month time period, women eligible for NHSBSP will be offered BC-Predict in four screening sites. Three screening sites will offer women usual NHSBSP. In the following 8-months the study sites offering usual NHSBSP switch to BC-Predict and vice versa. Key potential benefits including uptake of risk consultations, chemoprevention and additional screening will be obtained for both groups. Key potential harms such as increased anxiety will be obtained via self-report questionnaires, with embedded qualitative process analysis. A decision-analytic model-based cost-effectiveness analysis will identify the key uncertainties underpinning the relative cost-effectiveness of embedding BC-Predict into NHSBSP.

**Discussion:**

We will assess the feasibility of integrating BC-Predict into the NHSBSP, and identify the main uncertainties for a definitive evaluation of the clinical and cost-effectiveness of BC-Predict.

**Trial registration:**

Retrospectively registered with clinicaltrials.gov (NCT04359420).

## Background

Breast cancer is the most common cancer in the UK and a leading cause of death in women [1]. Each year, approximately 55,000 women are diagnosed with breast cancer, of whom approximately 11,400 will die from the disease [1]. Although deaths from breast cancer have been decreasing in many Western countries, the incidence of breast cancer is continuing to increase [2–4]. To identify breast cancer at an earlier and more treatable stage, nearly two million women are screened in the National Health Service Breast Screening Programme (NHSBSP) in England every year [5]. The NHSBSP currently invites women aged 50 to70 years (though some breast screening units are trialling screening from ages 47 to 73 years) for three-yearly mammograms. The NHSBSP also undertakes screening of very high-risk women with high-penetrance mutations in genes such as *BRCA1, BRCA2* and *TP53.* These women are offered annual Magnetic Resonance Imaging screening between ages 30 to 50 years and annual mammography between 40 to 70 years.

In 2013, the National Institute for Health and Care Excellence (NICE) recommended that women at high risk of breast cancer who are not high penetrance gene carriers (lifetime risk ≥30%, 10-year risk ≥8%), should be offered annual breast screening between the ages of 40 to 59 years; and those at moderate risk (lifetime risk 17–29%, 10-year risk 3–7.9% aged 40 years), should be offered annual mammography from 40 to 49 years [6], but considered for annual screening aged 50 to 59 years. NICE guidance also recommends that women at high risk of breast cancer are offered chemoprevention with tamoxifen, anastrozole or raloxifene (considered in moderate risk) and advice on weight control and physical activity [6]. So far, it is estimated that only about 1 in 6 women who are at high-risk as defined by NICE (≥8% ten-year risk of breast cancer) have been actively identified by attending Family History, Risk and Prevention (FHRP) Clinics [7, 8].

Risk stratification in the NHSBSP could identify many of the 5 in 6 women who are at high-risk but are not aware of this, as well as a larger number of women at moderate risk. It is possible to accurately estimate a woman’s individual risk of developing breast cancer through information on breast density derived from mammography and self-report questions assessing family history and factors affecting hormone levels, e.g. using the Tyrer-Cuzick algorithm [9]. A previous study (PROCAS) provided 10-year risk estimates to over 54,000 women in the NHSBSP in Manchester, England [10]. This study was the first time that personalised breast cancer risk estimates were calculated for large numbers of women from the general breast screening population. The PROCAS study found that at least 3% of women are high risk (≥8% 10-year risk) when all risk factors including mammographic density are assessed and a further 10% are at moderate risk (5–7.9% 10-year risk) [7, 8]. Given that only 0.5% of the population have identified themselves as high risk, this means that there are approximately an additional 450,000 women in England (aged 30 to 70 years) at high risk that NICE guidance indicates should be offered chemoprevention and annual mammography.

The introduction of risk stratification in the NHSBSP could allow the potential benefits of more frequent screening and/ or chemoprevention to be realised on a population basis, and potentially allow women at lower risk to have less frequent screening recommended. In principle, a risk-stratified NHSBSP should result in a better balance of benefits, harms and NHS costs and there is some emerging early evidence to support this premise [11]. The benefits might be fewer breast cancers due to chemoprevention, and reduced breast cancer mortality arising from NHSBSP detecting more breast cancers at an earlier and more treatable stage. There might also be grounds for reducing screening for women at lower risk, who would be less likely to develop high grade tumours [12]. Reducing screening in women at lower risk would produce fewer harms of screening in this group, such as fewer false positive test results in lower risk women [13].

The consequences of introducing risk stratified screening in the NHSBSP are unclear. In the PROCAS study, communication of risk estimates happened 3 to 5 years after women provided their questionnaire data and consent [10], so that study provides limited information about the consequences of receiving risk estimates: the main purpose of that study was to validate risk prediction algorithms rather than as a new screening service model [8]. It is likely that, if aware of their risks, a sizeable proportion of women at high/moderate-risk would opt for chemoprevention with anastrozole/raloxifene/tamoxifen [7, 8, 10, 14, 15], as well as extra mammography in high-risk women [8]. The overall net effect of chemoprevention and additional screening is likely to be beneficial from a reduction in breast cancer incidence and mortality. By contrast, there are also several possible harms that could be brought about by the receipt of risk estimates. Communicating personal risk information to women could induce undue anxiety and worry. Although the best available evidence suggests that this is unlikely, this evidence has limitations such as a long time-lag between women agreeing to risk assessment and receiving risk results [16].

In addition, the mere offer of risk stratified screening may have potential adverse effects. It is possible that by offering risk stratified screening as part of the NHSBSP, women are put off from attending screening and thereby receiving its benefits. Evidence from PROCAS [10] suggests this is unlikely. Furthermore, as with all screening programmes within the NHS it is important that patients are provided with the necessary information in order to possess the knowledge to make an informed personal decision about whether to attend screening, and any treatment options that follow from screening [17]. There is currently no clear evidence to indicate whether risk-stratified screening could result in more informed decisions or not [18].

A final important group of possible drawbacks of implementing risk stratification are the potential costs, both personal and financial, to implementing the communication of risk information on such a scale, including increased NHS staff workload and additional healthcare resources. Evidence is therefore required about the key drivers of the relative cost-effectiveness of communicating breast cancer risk estimates compared with current NHSBSP practice, understanding the key uncertainties in the current evidence base and potential value of future research [19]. Overall, it is imperative in order to highlight whether risk stratified screening will induce harms and if so, how they can be mitigated so as not to outweigh benefits, and allow more effective use of healthcare resources.

We have developed an automated system (BC-Predict) for offering an assessment of breast cancer risk to women when they receive their NHSBSP invitation, and generating letters to feedback this risk to women and relevant healthcare professionals. A development phase involved working with healthcare professionals that ensured that the care pathways were workable, and that informatics procedures functioned as intended. The patient information materials were co-produced with women who would be eligible for BC-Predict to promote good understanding and informed choices, and also minimise harms such as unnecessary worry.

In BC-Predict, risk estimation can be offered in real-time to women invited for breast screening via an online web system to allow consent and self-report measures to be provided. Risk assessment is based on self-report questions and breast density estimates automatically derived from mammography, and can also incorporate information from currently known breast cancer Single Nucleotide Polymorphisms (SNPs), derived from DNA contained in saliva samples. Women who receive a clear mammogram result are then sent a letter providing their 10-year breast cancer risk within 6 to 8 weeks after their mammogram. Thus all women will know their risks. Those women at moderate (> 5% but < 8% 10-year risk) or high (≥8% 10-year) risk are encouraged to attend a consultation at a FHRP Clinic, to discuss the offer of more frequent screening and chemoprevention.

Although developmental work has shown BC-Predict to function as intended, it would not be appropriate to implement a system such as BC-Predict outside of a research setting, given the uncertainties around potential benefits, possible harms and cost effectiveness [20]. It would not even be proportionate or ethical to conduct the required large-scale definitive evaluation of clinical and cost-effectiveness, as this which would require the participation of hundreds of thousands of women to have sufficient power to detect its effect on breast cancer incidence and stage. Therefore, in line with the MRC Framework for Developing and Evaluating Complex Interventions [21], the present research has the goal of identifying and resolving key uncertainties regarding the feasibility of integrating BC-Predict into the NHSBSP and assessing the feasibility of a definitive study to assess whether the intervention translates into measurable effects on breast cancer incidence and stage, and is a cost-effective use of NHS resources. The present research will therefore quantify key drivers of the relative cost-effectiveness of communicating breast cancer risk estimates compared with current NHSBSP practice, understanding the key uncertainties in the current evidence base and potential value of future research.

A particular concern during the development phase was that that women from low socioeconomic and minority ethnic backgrounds are less likely to attend for screening [22–24]. Commonly cited reasons include language barriers, cultural incongruences and lack of understanding and knowledge about screening [22, 25, 26]. It is not presently known whether the introduction of risk-stratified screening would exacerbate these issues further or lead to increased non-attendance. In developing BC-Predict, interviews with a cohort of British-Pakistani women from low socioeconomic backgrounds found that views toward risk-stratified screening are favourable. However, as with the present screening programme language barriers could still prevent access and reduce women’s ability to make informed decisions [27]. Given this, in the present study we will assess whether women from low socioeconomic status backgrounds are less likely to take up the offer of risk-stratified screening.

The overall aim of the present research will be to establish whether providing women eligible for NHSBSP with personalised breast cancer risk (BC-Predict) estimation is feasible, by (a) measuring important potential harms and benefits of BC-Predict, (b) identifying the key drivers of the relative cost-effectiveness of embedding BC-Predict into the NHSBSP, and (c) attempting to understand the key issues affecting implementation of BC-Predict as part of the NHSBSP. This overall aim will be met by evaluating the BC-Predict system in a 16-month study running within the Greater Manchester, East Cheshire and East Lancashire NHS breast screening programmes, with the following three overarching objectives:

### Quantifying important potential benefits, particularly

- Uptake of BC-Predict amongst women offered it
- Uptake of risk consultation (for those eligible)
- Uptake of chemoprevention (for those offered it)
- Uptake of additional mammography (for those offered it)

### Quantifying important potential harms, particularly

- Lower uptake of NHSBSP amongst women offered BC-Predict
- Increased worry about breast cancer
- Increased general state anxiety
- Less informed choices regarding screening uptake

### Quantifying indicative estimates of the NHS costs and patient consequences, including

- Effects on health status and healthcare costs
- Key drivers of the relative cost-effectiveness of embedding BC-Predict into the NHSBSP
- Extent of uncertainty in the current evidence-based
- The potential value of future research.

## Methods/design

### Study design

A non-randomised fully counterbalanced study design will be used, to include equal numbers of participants from all sites who will be offered NHSBSP and BC-Predict. Specifically, in the initial 8-month time period, four screening sites will offer women eligible for breast screening BC-Predict. Three screening sites will offer women usual care NHSBSP. In the following 8-month time period the study sites switch to offer the other intervention (NHSBSP rather than BC-Predict; and vice versa). This ‘counter-balanced’ design will allow estimates of effect to be obtained from both within-sample and between-sample analyses.

### Setting

Women will be recruited from seven sites within three NHS Breast screening programmes: three sites within the Greater Manchester programme (Withington Community Hospital, Oldham lntegrated Care Centre and the Trafford mobile screening van only), and two sites each based in the East Cheshire (Macclesfield District General Hospital and Stockport mobile breast screening van locations) and East Lancashire (Burnley General Hospital and East Lancashire mobile breast screening van locations) programmes. Women invited to screening in East Cheshire and Withington/Trafford in the first 8 months of the study will be offered BC-Predict and women in East Lancashire and Oldham offered screening as usual. After 8 months, BC-Predict will be offered to women in East Lancashire and Oldham, with women in Cheshire and Withington/Trafford offered screening as usual.

### Participants

Recruitment is over a 16-month period and sites will each be open to recruitment to BC-Predict for a period of 8 months. Two groups of women will be invited to participate in the study (a) women invited for first time screening (“prevalent screens”), and (b) women invited during the screening round within which they reach 60 years (“incident screens” i.e. women aged 57 to 63 years). Posters advertising the study will be displayed in each of the participating screening sites to increase awareness of the study.

We will include women who are invited for usual care (NHSBSP) at each site to compare with women offered BC-Predict; however NHSBSP women will not be consented to the study as controls, as their personal information will not be accessed. Instead, core outcome measures will be obtained in aggregated form. This will provide a comparison with uptake to these services in the BC-Predict arm. Posters at study sites will inform women being offered NHSBSP that they can request their data is not included in any analysis.

Inclusion criteria are that the participant: (a) is born biologically female; (b) is invited for first breast screening appointment (any age); is aged 57 to 63 years (only at East Cheshire and East Lancashire NHSBSP); and (c) is able to provide informed consent and complete a risk assessment questionnaire. Exclusion criteria are that the participant: (a) is born male; (b) previously had breast cancer; (c) had bilateral mastectomy; or (d) has previously participated in the PROCAS study [10].

### Procedure

**Fig. 1:**
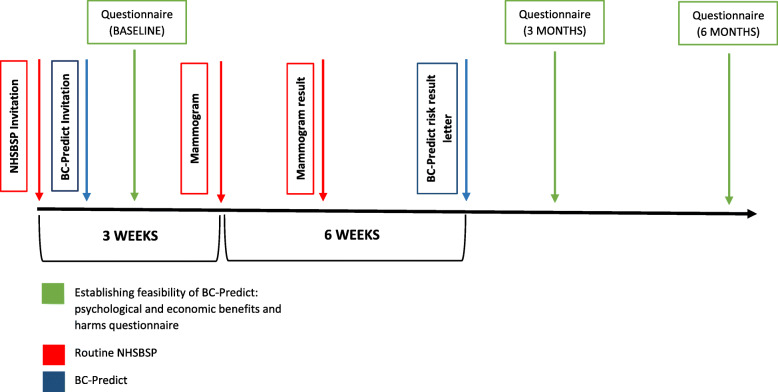
Timeline of Psychological-Impact study integrated with BC-Predict and NHSBSP

**Fig. 2:**
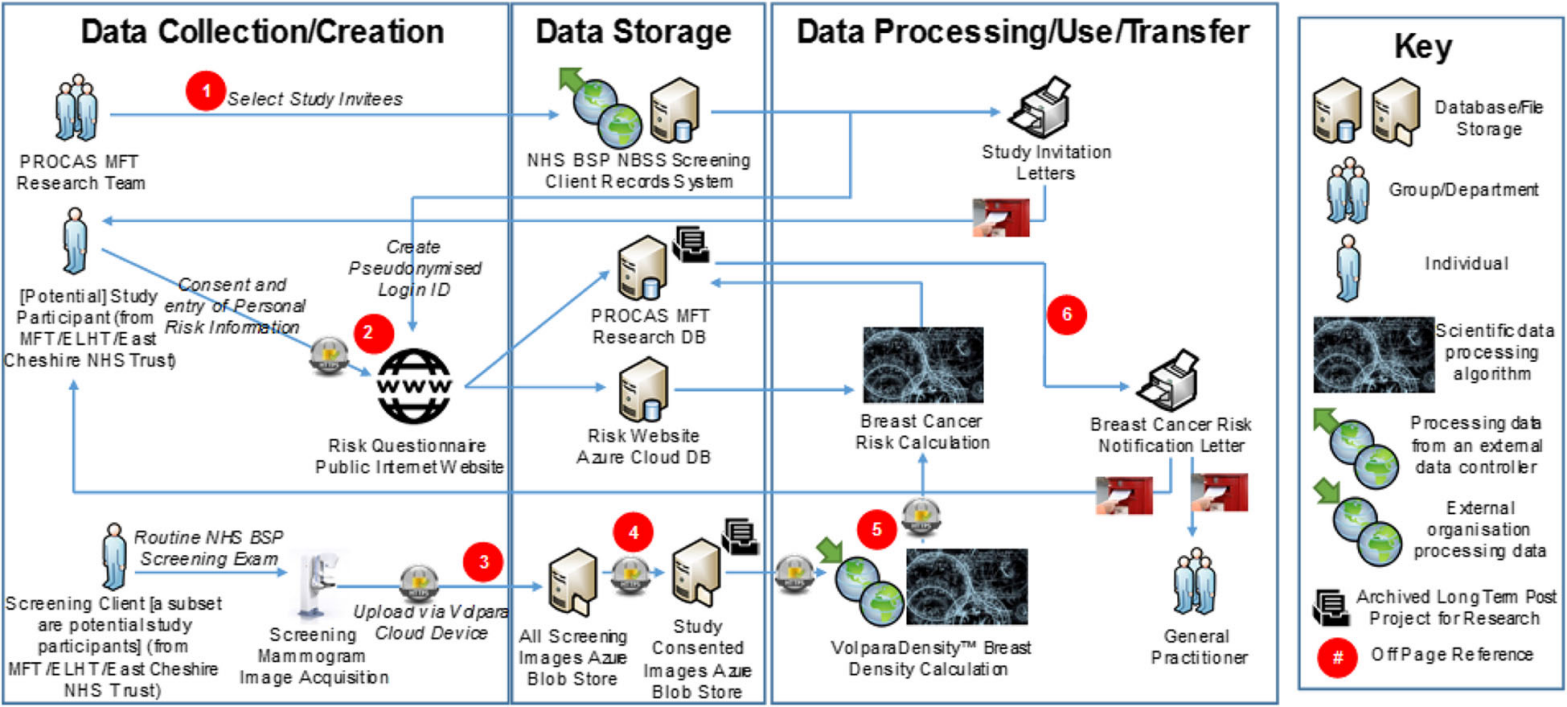
Study Participant Data Flow Diagram

Women being offered BC-Predict will be sent an invitation letter one to two working days after their breast screening invitation letter is sent. The BC-Predict invitation letter will be sent along with the participant information sheet and instructions directing prospective participants to the online risk assessment platform. Each invitation letter will include details of the participant’s “Date of first offered appointment”. This is the first breast screening appointment date that was offered to the participant. This date is of relevance because participants will be able to join the study either before the date of their first offered appointment or up to six-weeks after. After this time it will no longer be possible for them to login to the BC-Predict risk assessment platform. Prospective participants will be directed to telephone the study helpline if they have any questions, or if they require any further information prior to deciding whether or not to take part. The timeline from the participant perspective is shown in Fig. 1. An overview of data-flows is shown in Fig. 2.

Once participants have consented to the study online, they will be directed to the BC-Predict risk assessment questionnaire. Participants will be able to enter part of the questionnaire, save and return to it at a later date, as long as they do this within their six-week recruitment window. Assessment of the online questionnaire during the pilot phase estimated that most women would be able to complete this within 30 min. If a prospective participant doesn’t have access to the internet, a paper version of the questionnaire can be posted out to be completed along with a paper version of the consent form. The data recorded on the questionnaire will then be manually inputted into the online risk assessment platform by a member of the study team, and the standard process will be followed from this point.

Once a clear mammogram result has been provided, a risk feedback letter is generated based on the answers participants give in their questionnaire and mammographic breast density (calculated from uploaded raw data by Volpara systems). The percentage density is inserted into an online version of Tyrer-Cuzick v8 that includes an algorithm to adjust density for age BMI and menopausal status in into an odds ratio known as density residual [14]. The risk feedback letter will inform women that they are at “high” (≥8% 10-year risk), “moderate” (≥5% but < 8% 10-year risk), “average” (≥ 2% but < 5% 10-year risk), or “below average” risk (< 2% 10-year risk). Each letter will explain how the risk estimates were derived, and the implications of these. Each group of women will receive this letter in the post, along with a leaflet providing additional detail on breast cancer risk factors, signs and symptoms of breast cancer and how risk might be managed. Those women who complete the BC-Predict risk assessment questionnaire over the telephone will also be sent copies of this questionnaire so that they can check for data entry errors and the electronic consent form for participant’s records.

All BC-Predict participants will have been invited for breast screening but a proportion may choose not to attend their breast screening appointment; attendance at breast screening mammogram is not a compulsory part of the study. The Participant Information Sheet explains that participants’ mammographic breast density will be included in their risk assessment, providing they attend their mammogram within 6-weeks of their first offered appointment. It is also explained that including mammographic density increases accuracy of the risk assessment. Any participant who declines a mammogram or has a mammogram after this time will not have this data included in their risk assessment, which is explained in their risk feedback letter.

To assess self-reported harms and benefits, and to inform an economic analysis, a randomly selected subsample of *n* = 2108 women (*n* = 1054 each from usual care NHSBSP and BC-Predict) will be asked to complete questionnaires assessing psychological benefits and harms of BC-Predict at baseline, 3-months and 6-months. For women in both groups, the request to complete the questionnaire will be sent shortly after their mammography invitation but before their first offered mammogram appointment, asking for their help in evaluating a new approach to providing NHS breast screening. They will be given instructions to complete an online consent form and questionnaire using their unique study identification number on SmartSurvey (https://www.smartsurvey.co.uk/). The same women will be asked to complete the questionnaire three and 6 months after their first offered mammogram appointment. Women in both experimental groups will only receive follow-up questionnaires once they receive a clear mammogram result.

The risk assessment and feedback will take account of each patients’ journey through the NHSBSP. The study team will periodically check screening outcomes for participants. There are a number of initial screening outcomes: (a) clear mammogram and woman will be invited for routine breast screening in three-years (routine recall); (b) mammogram taken is technically inadequate so repeat mammogram is required (technical recall); (c): suspicious mammogram and further assessment required (recall for assessment). For all scenarios the GP will be informed of the participant’s involvement in the study and provided with their risk feedback.

Participants who are confirmed as having a routine recall screening outcome will receive their risk feedback after this, approximately 6 weeks after their mammogram. Participants who are invited for a technical recall/recall for assessment appointment, and attend this appointment within 6 months of their first scheduled technical recall/recall for assessment appointment, and who are subsequently confirmed as not having breast cancer will receive a risk feedback letter following confirmation of an absence of breast cancer. Participants who are invited for a technical recall or recall for assessment appointment but do not attend within 6 months of the first scheduled technical recall/recall for assessment appointment (i.e. those for whom there is no screening outcome within 6 months of initial screening outcome) will receive their risk feedback 6 months after joining BC-Predict.

Participants who do not attend a breast screening appointment within six-weeks of their first offered breast screening appointment will receive their risk feedback after this six-week period (i.e. 7 to 8 weeks since their first offered breast screening appointment).

Participants who are diagnosed with breast cancer will not receive a standard risk feedback letter. Participants will be sent a letter 1 year after diagnosis which will offer them feedback from the study. If they opt to receive this, they will be sent a personalised letter explaining their breast cancer risk factors.

### Measures

Two main types of measures will be used: core outcome measures and self-reported measures.

#### Core outcomes

The following nine core outcomes will be compared at 6-months post completion of recruitment for those offered BC-Predict and those offered usual care (NHSBSP):

1. Screening attendance at first offered screening episode.

2. Screening attendance within 180 days of episode opening.

3. Number of technical recalls.

4. Number of recalls for assessment.

5. Number of routine recalls.

6. Number of breast cancer diagnoses (and type/grade).

7. Subsequent consultation in FHRP clinics (and mode: telephone or face-to-face).

8. Subsequent enrolment for more frequent screening.

9. Subsequent prescription of chemoprevention. Data will be collected on each of the following aspects of this: (a) participant agrees/disagrees in clinic to take chemoprevention, (b) chemoprevention not appropriate, (c) chemoprevention appropriate but prescription not filled, (d) chemoprevention appropriate and prescription filled.

Data for the nine core outcomes will be collected for each consented BC-Predict participant by the research staff for the 6 months following participants’ mammography appointment. For these participants, information will be available directly from NHSBSP and FHRP clinic records. For those in the usual care arm of the study, anonymised data will be provided by NHSBSP and FHRP services, to provide overall numbers for each of the core outcomes. We will prospectively record any refinements to procedures, to allow examination of how these impact on uptake of services.

We will also assess uptake of BC-Predict, and examine variation by study site. Where changes to recruitment procedures are made, we will keep notes of this, and examine the effects of these changes on uptake of BC-Predict, to inform how risk stratified screening should be rolled out. We will also examine variations in uptake of services by Index of Multiple Deprivation deciles derived from postcode of women [28] invited for NHSBSP, to assess any potential exacerbation of health inequalities brought about by BC-Predict.

#### Self-reported outcomes

The self-reported measures of potential harms and benefits of BC-Predict to be completed by a sub-sample of participants are shown in Table 1.

**Table 1:** Self-reported measures to be assessed, at each of the three timepoints.

Baseline	3 months	6 months
State Anxiety [29]	State Anxiety [29]	State Anxiety [29]
Cancer Worry [30]	Cancer Worry [30]	Cancer Worry [30]
Risk perceptions [31]	Risk perceptions [31]	Risk perceptions [31]
Attitudes to screening [32]		Attitudes to screening [32]
Knowledge [33]		Knowledge [33]
Intention (future screening) [32]	Intention (future screening) [32]	Intention (future screening) [32]
Health status (EQ-5D5L) [34]	Health status (EQ-5D5L) [34]	Health status (EQ-5D5L) [34]
Capability [35]	Capability [35]	Capability [35]
	Satisfaction with information [36]	Satisfaction with information [36]

*Informed choices regarding screening will be estimated from attitudes to screening at baseline, knowledge and screening attendance, using a standard approach [17]

**Women invited to BC-Predict will receive the above. Women invited to NHS-BSP will receive the above minus the satisfaction with information questionnaire

### Analysis plan and power calculations

#### Core outcomes

In total, approximately *n* = 18,700 women will be offered BC-Predict and *n* = 18,700 will be offered usual care NHSBSP over the total 16-month period. Based on the recruitment rate in PROCAS, *n* = 18,700 women being offered BC-Predict should result in 8000 women taking it up. The attendance rate to usual NHSBSP in Greater Manchester is 69% [5, 10].

Core outcomes will be compared for cohorts of women who are invited to BC-Predict and those who are invited to usual care (NHSBSP). Thus we will have in excess of 8000 participants in BC-Predict and NHSBSP groups for both comparisons: (a) within-site and (b) between sites over the same time period. The primary outcomes are binary. Logistic regression will be the primary statistical analysis method. We shall assess heterogeneity effects using interaction tests in the logistic regression, to examine differences in outcomes by time or location or screening type (prevalent v incident). Even in the presence of geographic or temporal heterogeneity of the effect, or of carryover effects continuing beyond the crossover period, we will still have sufficient data for a valid and fully powered comparison.

For core outcomes 1 and 2, we are interested in equivalence, in that we anticipate that invitation to BC-Predict will not substantially affect screening attendance. With 18,700 women in each group we will have in excess of 90% power to establish equivalence, defined as a 95%CI on the difference which does not exceed ±5% on the attendance rate at first offered appointment, if the latter is around 50% [37]. Similarly, we will have more than 90% power for the same comparison for eventual attendance within 180 days if the latter is around 70%.

Arguably, the most difficult to collect core outcome is 9, the proportion taking up chemoprevention. On the basis of PROCAS results we would anticipate that 1169 of the 8000 who consent to BC-Predict would have sufficient risk to be considered for chemoprevention and that 10% of these would take it up [8, 38, 39]. Thus 117 of the 8000 women (1.5%) receiving the intervention might be expected to be prescribed chemoprevention. It is anticipated that very few in the 18,700 sent the standard screening invitation would be prescribed chemoprevention, but even if as many as 0.9% did so, we would have 90% power to detect this as significant at 5% level with two-sided testing, and 80% power if 10% took up chemoprevention. The Greater Manchester Medicines Management group has agreed a shared care protocol stating that the initial prescription of tamoxifen and anastrozole should be made by a FHRP specialist. As such data from even those in the control arm should be available from prescriptions made in the FHRP clinics.

#### Self-reported outcomes

Analyses will focus on comparisons between the responses of the BC-Predict and NHSBSP groups at 6 months follow up, controlling for baseline responses and baseline patient characteristics. We will use ANCOVA, first with baseline responses to the same questionnaires as covariates, secondly treating both baseline and 6-month responses as related endpoints, using hierarchical linear models. Out of the available self-reported outcomes, we have selected the primary outcome to be anxiety (State Trait Anxiety Inventory) at 6-months, but we will also examine effects on all measures included in Table 1, as well as effects at 3 months. We will use the variables concerning knowledge, and attitudes to screening, as well as screening attendance, to assess the extent to which decisions to attend screening are informed, in line with a standard approach to assessing this [17]. The measures of health status (EQ. 5D-5 level) and capability (ICECAP-A) will be converted to preference weights using published algorithms [40] and population tariffs [41], as appropriate.

In addition to providing information about potential harms of BC-Predict, secondary analyses will examine whether women who are randomised to receive questionnaires differ in terms of uptake of screening or BC-Predict. This will inform about the likelihood of biases being introduced by comparisons of questionnaire responses in a possible subsequent definitive trial.

The sample size calculation is based on the six-item short-form of the state scale of the State Trait Anxiety Inventory [29], which measures general anxiety currently experienced on a scale of 20 to 80. Previous research in England with women invited to breast cancer screening found a mean state anxiety score of 37 [42]. A score of 49 has been found in patients with a diagnosis of anxiety disorder [43].

Assuming a two-tailed independent samples t-test, then *n* = 1054 (*n* = 527 women per experimental group) will be required to have 90% power (with α = 0.05) to detect a small standardised difference of d = 0.2. This equates to a difference between adjacent response categories (e.g. “not at all” and “somewhat”) on 2.5 of the 20 items on the full form of the scale. We anticipate that asking 1054 women per group will result in responses from *n* = 527 women per group being obtained at both baseline and 6 months, assuming a 70% response rate on both rounds.

#### Economic analysis

An early economic analysis [44] will aim to identify the indicative estimates of the incremental costs and consequences and key drivers of the cost-effectiveness of a risk-stratified NHSBSP compared with the usual NHSBSP. A decision-analytic model-based cost-effectiveness analysis will capture the incremental NHS costs and consequences for a cohort of women eligible for NHSBSP in the UK over a life-time horizon. A decision-analytic model (a decision-tree combined with a published model) [11] will be structured to represent the care pathways of current NHSBSP practice (no risk feedback) and the proposed BC-Predict intervention in a sample of women eligible for the NHSBSP. The cost of the risk-stratified NHSBSP will be identified using a micro-costing study [45] and take account of the cost of the addition of SNPs to the risk estimation algorithm. The decision-tree will recognise the uptake of appropriate healthcare services (General Practice contact; FHRP Clinic referral and proportion of women starting chemopreventive medication e.g. anastrozole/tamoxifen/raloxifene). A published model [9] will be used to understand the lifetime impact on NHS costs and patient consequences of using different screening intervals based on risk-prediction, or usual NHSBSP. Using an economic model allows data assimilation from various sources (BC-Predict and systematic reviews; structured expert elicitation methods [46]) in a structured framework [47]. The model-base case analysis will focus on changes in health status (using EQ-5D-5 L) but explore the impact on capability (ICECAP-A) in a scenario analysis. These data will be obtained from the self-reported outcomes (health status (EQ-5D5L) [34]; capability (ICECAP-A) [35]) collected in the prospective study (see Table 1) and supplemented with published data to allow estimation of the impact on a life-time horizon. The EQ-5D5L [34] and ICECAP-A [35] have published preference weights that will allow calculation of quality adjusted life years for health and capability with and without the intervention. Parameter uncertainty in the decision-tree component of the model will be quantified using probabilistic sensitivity analysis for the base case analysis and scenario (capability) analysis. These two outcomes (health and capability) will then be used in two distinct value of information analyses (Expected Value of Perfect Information (EVPI) and expected value of partial perfect information (EVPPI)). The EVPI represents the maximum amount that should be spent on future research to gain perfect information to eliminate the possibility of a wrong (funding) decision. Further steps are then necessary to understand key parameters driving the uncertainty. This involves estimating the EVPPI that tells a decision maker which parameters are contributing to the uncertainty in the model and help to guide what type of additional evidence is most valuable.

### Three sub-studies

The present research also includes sub-studies. Although integral to the overall research, they are described here, to facilitate clear presentation of their aims and methods.

### Sub-study one: incorporation of SNP information into BC-predict risk estimates

#### Objectives

To determine uptake and acceptability of a DNA based risk estimate as part of routine NHSBSP appointments, and to quantify the higher proportions of women at high/moderate and lower risk obtained by adding SNP information.

#### Background

A subset of women will have the option to provide a sample of saliva from which DNA can be extracted. DNA will be extracted with standard techniques and currently known breast cancer SNPs associated with breast cancer typed. The results of this testing will be incorporated into the BC-Predict risk algorithm. Adding a genetic SNP score from a saliva sample to other risk factors not only potentially increases the accuracy of risk estimation, but also increases the discrimination of risk estimation, so that more women are identified as being at higher or lower risk, and fewer identified as being at population-average risk. It thereby increases the proportion of women identified at high-risk who can benefit from being offered NICE approved additional screening and drug prevention from 4 to 6%.

#### Methods

In total, it is expected that 1000 women will provide a DNA sample and receive a personalised breast cancer risk estimate incorporating their Polygenic Risk Score. All women invited for screening at Withington Community Hospital and Oldham Integrated Care Centre will be potentially eligible for the SNP sub-study, however, this will only be offered to women on a pragmatic basis depending on whether a member of staff is on site to assist with taking consent. A separate paper consent form will be completed by the participant in addition to the online consent form for the main study. Women giving their consent will be provided with an Oragene kit to place their salivary sample. They will be guided on site by a member of staff as to how to complete the sample.

#### Methods: data analysis

The proportion of women in the 1000 providing saliva DNA who are classified as NICE actionable high and moderate risk as well as below average risk will be compared to their classification without a SNP Polygenic Risk Score. Chi square statistics will compare the difference between risk categories with and without the addition of the SNP Polygenic Risk Score. These data will also be used in the proposed economic analysis.

### Sub-study two: understanding acceptability and implementation of BC-predict

#### Objectives

The main objectives are to explore service users’ views on acceptability of BC-Predict (interviews) and to assess the perceived impact of BC-Predict on the NHSBSP, FHRP Clinics and General Practice (focus groups).

#### Background

In addition to the quantitative measures of impact of BC-Predict, we will also carry out qualitative work as part of a process evaluation to understand the key issues behind successful implementation of the BC-Predict system [48]. The qualitative work will comprise one-to-one interviews with NHSBSP service users to explore acceptability of BC-Predict amongst women at varying levels of risk, where there is currently a dearth of evidence [49]. It will also employ focus groups with healthcare professionals to investigate the implementation, delivery and impact of BC-Predict on the current NHSBSP. This qualitative work will give insight into capacity issues, indication of the training and support required to deliver BC-Predict on a larger scale, as well as communication challenges and pathways for both service users and healthcare professionals. This will enable us to build an evidence base to inform practice and policy should BC-Predict be rolled out to the wider NHSBSP.

#### Methods

##### Patient interviews: design, sample, recruitment and data collection

A purposive sample of below average, average, moderate and high-risk women who had received BC-Predict will be invited to participate in a semi-structured interview. Below average and average risk women will be invited for interview 1 month after receiving their risk feedback letter. Moderate-risk and high-risk women will be invited for interview 6 months after receiving their risk feedback letter. This gives women in the moderate and high-risk groups the chance to explore extra screening options or medications prior to the interview. The BC-Predict online platform allows easy identification of women in each risk group in each location. We will aim to recruit up to 40 women to these interviews (up to 10 women per group) with variation in which study sites to which women were invited. In addition, questionnaire responses will guide sampling to allow variation in uptake of chemoprevention.

Data will be collected by semi-structured interview either face-to-face or over the phone, audio recorded and transcribed verbatim. The decision to stop recruitment will be based on whether the data collected is sufficient to answer the research questions and aims [50]. Therefore the depth of the data will be used as an indicator to cease recruitment. The decision to end recruitment will also be based on the active exploration of negative cases, as well as when there appears to be no new content being discussed in the final interviews of each risk group. All interviews will cover core issues including acceptability of BC-Predict and lifestyle modifications. Other issues will be covered are those that are most relevant to the risk estimate communicated, e.g. uptake of chemoprevention (e.g. GP advice) in higher risk women, and reassurance in below-average risk women. We will be sensitive to considering naturally occurring variation, e.g. women recruited differently due to SNP collection or across different study sites, or from diverse ethnic backgrounds.

##### Healthcare professional focus groups: design, sample, recruitment and data collection

General Practice, Radiology and FHRP Clinic staff will be invited to participate in focus groups 2 months after BC-Predict has stopped being provided in each location. The groups will examine how well prepared they and associated staff were for implementing BC-Predict, along with views on acceptability of BC-Predict and how its implementation could be facilitated when widely implemented. We will run a multidisciplinary focus group in each location (total sample = ~ 36). Focus groups will be audio recorded and transcribed verbatim. If a participant is unable to attend the focus group but would like to take part, they will be given the option to be interviewed face-to-face or over the phone.

The analysis of these data will also be used to generate a list of additional resources required and a quantitative estimate of the impact on resources such as staff time. The groups will also aim to estimate the approximate cost of providing the BC-Predict intervention. These estimates will inform the economic analyses.

#### Methods: data analysis

For both interviews and focus groups, data will be analysed using a manifest level approach to thematic analysis as the themes are likely to be predominately deductive. Thematic analysis seeks and reports the patterns inherent within the data collected. It is a common qualitative analysis method that results in a rich, complex, yet accessible account of the data [51]. Themes will be coded at the manifest (or explicit) level [52]. It will do so taking an essentialist approach, which means that we aim to report the experiences, meanings and the reality of the participants [53].

Coding will be conducted systematically and iteratively. Negative cases will be sought to test the emerging coding framework. Regular coding meetings will be held to refine the coding structure. Data will be coded by independent researchers to check reliability in qualitative methods and ensuring that the fit between data and analysis is maximised. Percentage agreement on presence will be calculated. Coding will continue until the team are satisfied that codes and themes adequately describe and capture the data. Data will be stored and organised within Nvivo software.

### Sub-study three: assessing feasibility of increasing screening interval for women at low risk

#### Objectives

To evaluate the impact of providing materials for women at low risk explaining that less frequent screening may provide a better balance of benefits and harms for them.

#### Background

Women at lower risk of breast cancer are likely to receive less benefit from the NHSBSP but are more likely to experience overdiagnosis and treatment for cancers that will not cause them harm if left untreated. In this sub-study, we have chosen the risk threshold of 1.5% or below in newly screened women over 10 years, as this is the average risk level for a 40-year old woman, who currently would not be screened for a further 10 years. (Note that in the rest of the PROCAS study, a threshold of 2% or below was used to indicate below-average risk [10], so we use the term “low” to distinguish this distinct threshold in the present research). PROCAS indicated that 13.5% of women screened have a 10-year breast cancer risk of less than 1.5% when assessed by Tyrer-Cuzick and mammographic density [10]. This group of women are at a lower risk of developing breast cancer and the tumours they develop are much more likely to be early stage and slow-growing [10, 12]. Existing data suggest that a risk stratified NHSBSP may not only be potentially cost-effective [11], but also that it may be potentially more cost-effective to delay screening in low-risk women by optimising the screen interval [54].

Nearly 90% of the population have indicated that “screening is almost always a good idea” [55] and many women would feel aggrieved if they felt they were being denied a service inequitably. Relatedly, attendance at screening provides reassurance and peace of mind [56], so a lack of screening may result in increased worry about breast cancer. However, this view may be partly due to a lack of general awareness of issues such as overdiagnosis [57]. Many national screening figures believe that less frequent screening for women at low risk may be an important component of risk-stratified screening. Further, our ongoing developmental work suggests that this idea is acceptable to many women, including women who have received a below-average risk estimates.

#### Methods: design, sample, recruitment and data collection

In the final 4 months of the 16-month period of implementation of risk estimation, we will extend the offer of risk provision to include information about how, for women at low risk of breast cancer delaying further NHSBSP for a further period of 5 years may provide a better balance of benefits and harms for them. This information will be presented to all women in East Lancashire and Oldham as part of the invitation process, and repeated for women at low risk (< 1.5% over 10 years) as part of their risk feedback letter and accompanying leaflet. Every woman identified as being at low risk will be asked to complete the online questionnaire assessing harms and benefits of BC-Predict at 6 months, in contrast to the main BC-Predict study, where only a sub-sample will be asked to complete this questionnaire.

#### Methods: data analysis

The primary outcome for this sub-study will be intentions to take up screening in 3 years in women assessed 6 months after being told that they are at low risk and receive the recommendation to delay attending screening. Our main focus will be on estimating the proportion of women intending to take up screening, but we will also formally compare this proportion with women who receive provision of “below average risk” breast cancer risk estimates (< 2%) but no screening recommendations, recruited over the previous 12 months of BC-Predict. Intention to attend screening is a consistent predictor of subsequent screening attendance (*r* = + 0.42 in a systematic review that identified k = 19 such tests) [58].

#### Qualitative process analysis

A qualitative process analysis will be conducted in line with MRC guidance [48]. A sample of low risk women (up to *n* = 12) who had received low risk estimates will be interviewed 1 month after receiving the feedback letter. They will be sample purposively to provide variation in the three screening sites (Oldham Integrated Care Centre, Burnley General Hospital and East Lancashire mobile breast screening van). Interviews will focus on the extent to which the possibility of receiving an estimate of low risk was considered before consenting to risk estimation, the acceptability of the information communicated and particularly the recommendation to delay screening, and any deliberations about delaying screening. Data will be analysed using a manifest, inductive thematic analysis, and will involve comparison of interviews with those women told they are at below average risk, but given no particular recommendation.

#### A decision analytic model-based economic analysis

A decision analytic-model based economic analysis will be used to understand the potential relative cost-effectiveness of using a modified screening interval for women identified to be at low-risk of breast cancer as part of a stratified-BSP compared with the current NHSBSP. This analysis will build on an early economic analysis we previously conducted [11]. The model will be populated with data from the published literature and the current study to understand the relative costs and benefits of the modified screening interval for low-risk women assuming the perspective of the NHS and the impact on QALYs over the lifetime horizon for the defined population of women eligible for NHSBSP. Extensive sensitivity analysis will be used to understand the key drivers of relative cost-effectiveness when implementing a modified screening interval for low-risk women.

## Discussion

The present research aims to provide evidence on the feasibility of risk-stratified screening, by providing information about likely effects, both positive and negative, and which of these effects are likely to drive cost effectiveness. Due to the wish to avoid participant burden, some additional potential benefits and harms of screening were not examined, and merit consideration in future research.

One key issue that the present research does not cover relates to the possible benefit that risk estimation may prompt women to consider changes in their health-related behaviours to reduce cancer risk. An estimated 20–30% of breast cancer cases are thought to be attributable to excess weight, weight gain lack of physical activity (PA) and high alcohol intakes [59–61]. In general, communicating personalised risk in the absence of supportive programmes has small effects on increasing healthy lifestyle behaviours that are not maintained [62, 63]. Nevertheless, studies that have used personalised risk communication to bring about changes in health-related behaviours to date have not used additional strategies to optimise behaviour change for which there is good evidence [63]. Further, even small effects on these behaviours are achieved by communicating personalised risk information, then large population reductions in these unhealthy behaviours should follow.

The BC-Predict feedback materials include information on which behaviours are likely to reduce breast cancer risk, but the programme does not include any attempts at promoting health-related behaviour change. Women at higher breast cancer risk will have a greater proportional risk reduction through following healthy lifestyle recommendations [64, 65]. There is evidence that these women may also be more motivated to initially engage with evidence-based behaviour change programmes, maintain engagement, and thereby produce more behaviour change [66]. By contrast, it is possible that the provision of low risk results to women my produce false reassurance. This could result in women at low risk being less inclined to engage in behaviours likely to promote health although the wider evidence suggests that is not likely [67].

The present research will provide key information on feasibility of implementing risk-stratified screening into routine breast cancer screening. It complements two large ongoing trials. The WISDOM trial in the USA [68] and the MyPeBS trial in several European countries [69] are designed to show that risk-stratified screening is non-inferior to routine breast cancer screening, in terms of the number of late-stage cancers detected. In particular, the present research does not focus on effectiveness, but instead will provide information about the likely harms and benefits of risk-stratified screening, and will identify what are the key uncertainties that are likely to inform effectiveness and cost-effectiveness. It also has a more pragmatic focus than these two large ongoing trials, in considering what are the likely effects on the healthcare system when implementing risk stratification as part of routine NHSBSP, including an explicit quantitative and qualitative process analysis of the effects of this implementation.

## Data Availability

Not applicable, as protocol paper.

## Abbreviations

EVPI: Expected value of perfect information
EVPPI: Expected value of partial perfect information
FHRP: Family history, risk and prevention
NHSBSP: National health service breast screening programme
NICE: National institute for health and care excellence
PROCAS: Predicting risk of cancer at screening study
QALY: Quality-adjusted life year
SNP: Single nucleotide polymorphisms
